# Parafoveal processing and transposed‐letter effects in dyslexic reading

**DOI:** 10.1002/dys.1721

**Published:** 2022-07-11

**Authors:** Julie A. Kirkby, Rhiannon S. Barrington, Denis Drieghe, Simon P. Liversedge

**Affiliations:** ^1^ Department of Psychology, Faculty of Science and Technology Bournemouth University, Fern Barrow Poole Dorset UK; ^2^ School of Psychology University of Southampton, University Road Southampton Hampshire UK; ^3^ School of Psychology and Computer Science University of Central Lancashire Preston Lancashire UK

**Keywords:** dyslexia, eye movements, parafoveal processing, reading, transposed‐letter effects

## Abstract

During parafoveal processing, skilled readers encode letter identity independently of letter position (Johnson et al., 2007). In the current experiment, we examined orthographic parafoveal processing in readers with dyslexia. Specifically, the eye movements of skilled readers and adult readers with dyslexia were recorded during a boundary paradigm experiment (Rayner, 1975). Parafoveal previews were either identical to the target word (e.g., *nearly*), a transposed‐letter preview (e.g., *enarly*), or a substituted‐letter preview (e.g., *acarly*). Dyslexic and non‐dyslexic readers demonstrated orthographic parafoveal preview benefits during silent sentence reading and both reading groups encoded letter identity and letter position information parafoveally. However, dyslexic adults showed, that very early in lexical processing, during parafoveal preview, the positional information of a word's initial letters were encoded less flexibly compared to during skilled adult reading. We suggest that dyslexic readers are less able to benefit from correct letter identity information (i.e., in the letter transposition previews) due to the lack of direct mapping of orthography to phonology. The current findings demonstrate that dyslexic readers show consistent and dyslexic‐specific reading difficulties in foveal and parafoveal processing during silent sentence reading.

The purpose of the current study was to examine orthographic parafoveal processing in readers with dyslexia. Specifically, we compared dyslexic and non‐dyslexic readers on their ability to encode letter identity and letter position in the parafovea. Dyslexic adult readers were compared to skilled adult readers to determine whether readers with dyslexia independently encode letter position information during parafoveal processing in a similar manner to those without dyslexia.

Skilled reading relies upon readers correctly and rapidly identifying phonological information from the orthographic form. Thus readers need to be able to sufficiently allocate their attention to identify the orthographic properties of a word, such as letter identity and letter position, and then determine the correct phonological mapping for that letter or combination of letters. Indeed, letter identity and letter position encoding are fundamental processes in visual word recognition that allows readers to distinguish the phonological output of anagrams such as *was* and *saw*.

Whilst letter position is an important aspect of visual word recognition, it is encoded with a level of flexibility, for skilled adult readers. Studies of single word recognition in skilled adult readers (Chambers, 1979; Christianson, Johnson, & Rayner, 2005; Forster, Davis, Schoknecht, & Carter, 1987; O'Connor & Forster, 1981; Perea & Fraga, 2006; Perea & Lupker, 2003a, 2003b, 2004) have found that nonwords with transposition of two letters (e.g., *jugde*), where the nonword unit contains the same orthographic content as the base word, are more similar (e.g., *judge*) than nonwords with two substituted letters (SLs) (e.g., *jupte*). This is known as the transposed‐letter (TL) effect and indicates that words with TLs significantly activate the lexical representation of the base word more than words with SL. Therefore, letter identity encoding is not specific to letter position; letter identity and letter position are encoded independently which results in flexible letter position encoding.

The TL effect has challenged traditional models of visual word recognition. According to models such as the Interactive Activation model (McClelland & Rumelhart, 1981); the Dual Route Cascaded model (Coltheart et al., 2001); the Multiple Read Out model (Grainger & Jacobs, 1996); the Activation‐Verification model (Paap et al., 1982), and the Parallel Distributed Processing model (Harm & Seidenberg, 1999), a transposition (where two letters change positions) should be just as disruptive as a double letter substitution. However, models such as the SOLAR model (Davis, 1999, 2010); the Open Bigram model (Grainger & van Heuven, 2003; Grainger et al., 2006); the Overlap model (Gómez, Ratcliff, & Perea, 2008); and the SERIOL model (Whitney, 2001) incorporate more flexible mechanisms to encode letter position information and can account for the independent encoding of letter identity and position information.

The overlap model (Gómez et al., 2008) assumes that when the nonword is presented briefly, the position that corresponds to each letter in the sequence is not precisely encoded and as a result, the visual information which corresponds to each letter is distributed over the entire word space (position uncertainty assumption). Other models, such as the Open Bigram (Grainger & van Heuven, 2003; see also Grainger et al., 2006) and the SERIOL model (Whitney, 2001) assume that letter position is encoded through contextual information.

While these recent models of word recognition (e.g., Davis, 1999, 2010; Gómez et al., 2008; Grainger & van Heuven, 2003; Whitney, 2001) are useful in explaining flexible letter position encoding foveally, they have not yet been extended to consider flexible letter position encoding during parafoveal processing. They do, however, posit that both orthography and phonology might contribute to lexical identification (e.g., Coltheart et al., 2001; Grainger & Ziegler, 2011; Perry et al., 2007) and provide insight into pre‐processing of the upcoming word *n* + 1. Grainger and Ziegler proposed that both phonological and orthographic characteristics of lexical stimuli exert an influence on lexical identification via two processing routes. A coarse‐grained processing route and a fine‐grained processing route. The coarse‐grained route permits direct semantic access from orthographic form and allows a level of flexibility in orthographic encoding. The fine‐grained route allows access to semantics via commonly co‐occurring letter patterns being processed and mapped onto their corresponding phonological representations with less encoding flexibility. Skilled readers show a decreased reliance on phonological decoding (serial sounding out of letter sounds) and an increased reliance on coarse‐grained processing. However, the fine‐grained processing still occurs and allows phonological recoding. There is evidence for fine‐grained processing in adults, teen readers, and children as young as 8 years old (Blythe et al., 2018, 2020; Milledge et al., 2021; Pollatsek et al., 1992), where skilled readers have been shown to pre‐process phonology from word *n* + 1 (phonological recoding) and, importantly, this was modulated by orthographic similarity.

A body of empirical work has demonstrated TL effects during parafoveal processing during silent sentence reading. This work typically used the boundary paradigm (Rayner, 1975) to explore parafoveal preview benefits during reading (see Schotter, Angele, & Rayner, 2012 for a review). In the boundary paradigm (Rayner, 1975) an invisible boundary is placed between a pre‐target and target word. As long as the reader's gaze is left of the boundary the target word is replaced by a preview such that this preview may share all the properties of the target word, that is, an identity preview, or very few properties of the target word, that is, an unrelated word or random letter manipulation. When the reader's eyes move across the boundary, the preview word changes to the actual target word. This change occurs during a saccade, during which saccadic suppression prevents the intake of additional visual information, so the reader does not usually notice the change. A parafoveal preview benefit is apparent from shorter fixations on the target word when the parafoveal preview is identical to the target, compared to when the parafoveal preview is not identical.

Using the boundary paradigm (Rayner, 1975), Johnson, Perea, and Rayner (2007) showed that the identity preview (IP) condition (i.e., *judge*) provided the greatest benefit, however, TL previews (e.g., *jugde*) provided more benefit than SL previews (e.g., *judpe*). This pattern of results indicated that although correct letter position facilitates the word retrieval processes, letter identity information is encoded independently to letter position. Thus, during parafoveal processing, letter position is encoded flexibly where readers encode letter position parafoveally in a similar manner to that shown during foveal word processing.

Transpositions in word‐initial letters may play an important role in visual word recognition due to their spatial location and/or due to the fact that initial letters of a word are intrinsically more important for lexical identification. Empirical evidence from isolated word recognition has shown that manipulations involving the first letter of the word do not cause a TL effect. That is, nonwords with TLs (demula‐MEDULA) were equally effective primes for the base word as were nonwords with two SLs in the same positions (berula‐MEDULA; e.g., Perea & Lupker, 2004, 2007; Schoonbaert & Grainger, 2004). It may be that letters within a word are processed from left to right during lexical identification and as a consequence, these initial letters are central to allowing the reader to determine the identity of that word. White, Johnson, Liversedge, and Rayner (2008) explored these possible explanations using the moving window paradigm (McConkie & Rayner, 1975) to prevent readers from gaining parafoveal preview benefit whilst they read sentences with TLs manipulations. The moving window paradigm uses a saccade‐contingent change technique (McConkie & Rayner, 1975; Rayner, 1975) in which all words to the right of fixation have letters replaced with *X*s. Therefore, words to the right of fixation were only made available once the eye moved to them, ensuring that all words were processed exclusively in foveal vision. White et al. (2008) found that, even when readers had no parafoveal preview of the upcoming words, transpositions within the initial two letters of a word still caused greater disruption to lexical processing than internal transpositions. The authors, therefore, concluded that the importance of the initial letters of a word does not occur as a consequence of the spatial location of the letters (i.e., parafoveal preview of initial letters being visually clearer than those of internal letters), but rather due to some intrinsic importance of the initial letters in regard to lexical identification.

The intrinsic importance of the initial letters may be related to the need for sequential activation of phonological codes, particularly in determining the phonological onset of the word. Research has demonstrated that phonological information is encoded before lexical access (for a review see Leinenger, 2014) whilst orthographic codes appear to be activated slightly earlier than phonological codes (Lee, Rayner, & Pollatsek, 1999), phonological information clearly still impacts lexical activation. Milledge et al. (2021) suggested that the first letters of an upcoming word in the preview are critical with respect to phonological pre‐processing. Moreover, the findings indicated that the orthographic similarity, of pseudohomophone previews and target words was important, particularly for children compared to skilled adult readers. Past research has shown that the first letter plays a vital role in skilled adult readers' ability to lexically identify a word, both under direct fixation and, critically, during parafoveal pre‐processing (e.g., Briihl & Inhoff, 1995; Inhoff, 1987, 1989; Johnson & Eisler, 2012; Johnson et al., 2007; Milledge et al., 2021; White et al., 2008).

Johnson et al. (2007) manipulated the parafoveal preview of internal versus final letters (Experiment 2) and initial versus final letters (Experiment 3). They found a TL effect for internal letters but not for final letters (Experiment 2). In Experiment 3, they found a TL effect for initial letters in gaze duration (reliable only by participants, not by items) and a nonsignificant, numerical tendency of about 10 ms in both first and single fixation duration.

Furthermore, Pagán, Blythe, and Liversedge (2016) explored the TL effect, in initial letters, during parafoveal processing for adults and typically developing children. They found that TL effects did occur in the initial trigram of the parafoveal word—in both adults and typically developing children. Specifically, they found that the TL effect occurred for transpositions and substitutions in the first two letters of the parafoveal word (letter positions 1 and 2; acptain‐imptain when the base word is captain) and for the second two letters of the parafoveal word (letter positions 2 and 3; cpatain‐cgotain). There was, however, no TL effect when transpositions occurred for non‐adjacent letters (i.e., letters 1 and 3; pactain‐gartain).

While there have been numerous studies into parafoveal processing for skilled adult readers (for a review see, Schotter et al., 2012), there is still a paucity of research exploring parafoveal processing in dyslexic readers, particularly during reading. There are, however, studies that have found evidence for parafoveal processing for dyslexic readers during Rapid Automized Naming (RAN, Wolf & Denckla, 2005; see Jones, Ashby, & Branigan, 2013, and Yan, Pan, Laubrock, Kliegl, & Shu, 2013). Jones and colleagues published a body of work that demonstrated that whilst adult dyslexic readers do make use of parafoveal information during RAN, parafoveal information is a potential source of confusion (Jones, et al., 2010; 2008; 2013). Specifically, Jones et al. manipulated whether the parafoveal information available to readers in position *n* + 1 was orthographically or phonologically similar to the information presented in position n (e.g., p–q; b–d). They used a contingent change paradigm so that potentially confusable letters were presented only parafoveally: When the participant fixated the *n* + 1 location the similar or dissimilar preview was replaced by a different letter. They found that dyslexic readers were more susceptible to orthographic confusability in the parafovea. Jones et al. (2013) suggested that not only is there interference from orthographic parafoveal information for dyslexic readers but also a lag in the effect from the interference that indicates slower parafoveal processing for readers with dyslexia. The authors concluded that the confusability found in readers with dyslexia may result from degraded orthographic as well as phonological representations, making it more difficult for dyslexic readers to distinguish between, and thus slower to select and retrieve, the correct orthographic and phonological representations.

Further to Jones et al. (2013), Silva et al. (2016) used a naming task to manipulate the parafoveal load and the parafoveal preview potential of each item. Silva et al. explored the parafoveal load cost (increased processing time for the fixated item *N*, when followed by item *N* + 1 in the parafovea) and the parafoveal preview benefit (decreased processing time for item *N* when followed by item *N* − 1 in the parafovea). Although comparisons between dyslexic and non‐dyslexic readers did not yield group differences in parafoveal load costs, they did find evidence of parafoveal preview benefits for adult dyslexic readers (albeit reduced).

Using the boundary paradigm with identity previews (IP; e.g., *nearly*), TL previews (TL; e.g., *enarly*), and SL previews (SL; e.g., *acarly*) the current research aimed to clarify (a) whether adults with dyslexia exhibit parafoveal preview benefit during silent sentence reading and (b) to explore the extent to which the TL effect is evident in dyslexic reading in comparison to skilled adult readers. Based on the studies described above, we predicted that readers with and without dyslexia would show parafoveal preview benefits during reading. Based on the findings of Jones et al. (2013), we predicted that dyslexic readers would show parafoveal preview benefits at a delayed timeframe compared to skilled adult readers.

In relationship to the orthographic preview manipulations, we predicted that similar to previous work (e.g., Johnson et al., 2007; Pagán et al., 2016; Tiffin‐Richards & Schroeder, 2015), skilled readers will demonstrate the typical pattern of effects whereby preview of the identity condition would lead to shorter viewing durations than the TL and SL previews once the target word was fixated. Facilitatory effects are suggested to be a function of both the orthographic and phonological overlap of the preview and the target word (e.g., Blythe et al., 2018, 2020). In skilled adult reading, orthographic codes are activated slightly earlier than phonological codes (Lee et al., 1999). Furthermore, the TL condition would lead to shorter viewing durations than the SL preview when the target word was fixated. Skilled reading relies on fast orthographic encoding, and potentially, this means that the benefit of TL previews is increased for skilled adults compared to dyslexic readers. Therefore, we predicted that dyslexic readers would demonstrate a smaller TL effect compared to that skilled adult readers. And for dyslexic readers, there would be an increased benefit for identity previews compared to TL previews.

In addition to understanding parafoveal eye movement patterns, we aimed to fully document the global eye movement behaviour of dyslexic readers and compare those to skilled readers. Any differences in eye movement behaviour between these two groups of readers will further our knowledge of the condition and aid in documenting the difficulty experienced by dyslexic readers in linguistic information processing.

## 1 METHOD

### 1.1 Participants

Twenty‐five university students were diagnosed with developmental dyslexia (mean age of 21 years and 6 months and SD: 4 years and 6 months) and 26 university students without dyslexia (mean age 20 years 3 months and SD: 1 years and 3 months) participated in the experiment. Students with dyslexia had a prior, independent diagnosis of dyslexia and diagnosis was further supported by deficits in standardized tests of reading ability and RAN (see Results section). All were native English speakers with normal or corrected to normal vision and were recruited from Bournemouth University. All participants performed within the normal range on a standardized intelligence test (IQ ≥ 90).

### 1.2 Apparatus

Eye movements were recorded from the right eye using an SR Research Eyelink 1,000 eye‐tracker. Sentences were presented at a viewing distance of 660 mm on a 21 in. Formac ProNitron 21/750 monitor with a screen resolution of 1,024 × 768 pixels and a refresh rate of 120 Hz. Sentences were presented in black 14 pt Courier New font on a white background. At this viewing distance, 3 characters equalled 1° of visual angle.

### 1.3 Design and stimuli

Three parafoveal preview conditions were presented using the boundary paradigm (Rayner, 1975). Parafoveal previews of the target words were either (a) identity preview, (b) a TL nonword, or (c) a SL nonword (See Table 1 for an example). The manipulation occurred in the initial two letters of the target word. For the TL conditions, the positions of the two initial letters were switched, and for the SL conditions the initial two letters were replaced with visually similar letters (ascenders were replaced with ascenders and descenders with descenders). Target words were always 6‐letter words and preceded by a 5‐ or 6‐letter pre‐target word. Pre‐target and target words were presented around the middle of the sentence and were high‐frequency words. The mean frequency of the pre‐target word was 535 counts per million and the mean frequency of the target word was 262 counts per million (BNC; British National Corpus).

**TABLE 1:** Examples of the target word manipulation

	Example sentence
Identity preview	During the earthquake the table *nearly* collapsed on him.
Transposed‐letters	During the earthquake the table *enarly* collapsed on him.
Substituted‐letters	During the earthquake the table *acarly* collapsed on him.

*Note*: Sentence frames included either an identity preview, a transposed‐letter preview, or a substituted‐letter preview.

The stimuli consisted of 90 sentence frames. For each sentence frame, there were 3 versions corresponding to the three parafoveal preview conditions. Three experimental lists were constructed whereby each list contained a different version of each sentence frame and the parafoveal preview manipulations were randomized across the 3 experimental lists so each participant saw 30 sentences from each of the three preview conditions.

The eye contingent change boundary was located at the end of the pre‐target word and to the left of the space preceding the target word. When the eyes moved past the invisible boundary, the target word changed from the parafoveal preview to the target word. The correct target word then remained in the sentence throughout the remaining duration of the trial. Display changes were typically undetected by the readers as they occurred during a saccade (when visual information is suppressed). Indeed, when participants were questioned as to whether they noticed anything unusual during the experiment, very few reported noticing anything, and those who did notice something suggested it occurred in a very small number of trials (less than 4 trials) and were unable to explain what had happened.

### 1.4 Offline measures of reading ability and IQ

All participants completed a range of offline tests. IQ was measured using two subtests of the Wechsler Abbreviated Scale of Intelligence (WASI; Wechsler, 1999): (a) the vocabulary subtest and (b) the matrix reasoning subtest. The Test of Word Reading Efficiency (TOWRE; Torgesen, Wagner, & Rashotte, 1999) was conducted to provide information on the participants' reading ability. All participants completed the Number and Letters measures of the Rapid Automatized Naming test (RAN; Wolf & Denckla, 2005).

### 1.5 Procedure

Participants sat in front of a computer screen with their heads positioned in a forehead restraint and chin rest to minimize head movements. They were instructed to read the sentences silently for comprehension and to press a button on a gamepad once they had finished reading. A 3‐point calibration was conducted prior to the experimental trials; an accurate calibration was accepted when the average errors in the validation were below 0.3° of visual angle. Calibrations were confirmed throughout the experiment and repeated when required. Each trial began with a gaze‐contingent box (a small black square) presented on the left‐hand side of the screen, positioned so that the initial letter of the sentence occupied the same location. Once the participant had fixated the square for 250 ms, the sentence appeared on the screen. Participants then read the sentence silently and terminated the trial with a button press. After 25% of the experimental sentences a “yes/ no” comprehension question appeared; participants were required to press a corresponding button to answer the question. The mean accuracy in comprehension score was 95.83% correct for dyslexic readers and 96.82% for skilled adult readers. There was no significant difference in the accuracy scores for comprehension for the two reading groups, |*t*(49)| < 1, n.s.

### 1.6 Statistical analysis

Prior to the analysis, fixations less than 80 ms were either merged into nearby longer fixations or excluded and fixations more than 800 ms were excluded from the data set (5.07% of fixations). Additional trials were excluded based upon the following criteria: (a) when the boundary was triggered prior to a saccade being made across the boundary, (b) when the display change was completed more than 10 ms after a fixation landing on the target word, (c) when the end of a saccade briefly crossed the boundary but the successive fixation remained in a position before the boundary, (d) when participants blinked on either the pre‐target or target word, and (e) when the participants skipped either the pre‐target or target word. In total 1,937 trials were removed from the analyses (31.66% of the dataset), data were excluded similarly across groups and conditions.

## 2 RESULTS

Analyses were conducted for both global and local measures. Global measures refer to results from all of the fixations within the sentence whereas local measures were based solely on the eye movements that occurred on the target word. Data were analysed using linear mixed models (LMMs) using the lme4 package (version 1.1–20) in R (version 3.5.2). For global analyses, the reading group was the fixed factor for all models. For local analyses, both the reading group and preview condition were fixed factors for all models. Participants and items were specified as random effects for both global and local analyses. For each dependent measure, a “full” random structure was implemented including all varying intercepts and slopes of the main effects and their interaction (maximal random effects structure as suggested by Barr, Levy, Scheepers, & Tily, 2013). If the “full” model failed to converge, or there were too many parameters to fit the data (as indicated by nearly perfect or perfect correlations of 0.99, 1, −0.99, or −1 in the random structure), the random structure was systematically trimmed (first by removing interactions between slopes for random effects, and if necessary also by removing correlations). Given our specific predictions, successive difference contrasts were used for the preview condition (comparing identity previews and TL previews, followed by TL previews and SL previews). Treatment contrasts were used for the Reading group with Skilled Readers set as the baseline. For each contrast, we report beta values (*b*), standard error (SE), and *t* or *z* statistics. Fixation time analyses were carried out on log‐transformed models to increase normality and count data were analysed using generalized LMMs following a Poisson distribution.

### 2.1 Off‐line measures

Mean scores and statistical analyses for the offline tests are presented in Table 2. There were no significant differences in the IQ scores of the two groups. However, the adults with dyslexia scored significantly lower on the TOWRE (Torgesen et al., 1999) compared to the skilled adult readers. Scores for both the number and letter subsets of the RAN (Wolf & Denckla, 2005) were also significantly lower for adults with dyslexia compared to skilled adult readers.

**TABLE 2:** Mean scores and statistical analyses for the offline tests for adults with and without dyslexia

	Dyslexic readers	Skilled readers	*t*‐test result
IQ	105.40 (7.92)	108.08 (6.36)	*t*(49) = −1.33, *p* = .188
TOWRE	82.60 (11.43)	102.15 (12.67)	*t*(49) = −5.78, *p* < .001
RAN numbers	103.92 (3.87)	111.38 (3.81)	*t*(49) = −6.94, *p* < .001
RAN letters	101.16 (5.94)	109.12 (4.59)	*t*(49) = −5.36, *p* < .001

*Note*: Standard scores are provided for IQ, reading ability (measured via the TOWRE) and RAN numbers and letters. Standard deviations are shown in parentheses.

### 2.2 Global measures

The following global measures were included; total sentence reading time, average saccade amplitude (measured in character spaces), average forward and regressive fixation duration, and the total number of forward and regressive fixations per sentence (See Tables 3 and 4 for model outputs). Forward and regressive fixations were classified based upon the previous saccade direction (fixations preceded by a rightward saccade are considered forward fixations and fixations preceded by a leftward saccade are referred to as regressive fixations).

**TABLE 3:** Model output for LMMs was conducted for global reading measures of total reading time (ms), saccade amplitude (degrees), average forward fixation duration (ms), and average regressive fixation duration

	Total time		Average saccade amplitude			Forward fixation duration			Regressive fixation duration			
	*b*	SE	*t*	*b*	SE	*T*	*b*	SE	*t*	*b*	SE	*T*
Intercept	7.89	0.04	**181.30**	0.99	0.04	**26.18**	5.37	0.02	**268.97**	5.27	0.02	**257.67**
Dyslexic readers	0.40	0.06	**6.57**	−0.07	0.05	−1.26	0.08	0.03	**2.80**	0.11	0.03	**3.83**

*Note*: Significant *t* values (|t| ≥ 1.96) are marked in **bold**.

**TABLE 4:** Model output for GLMMs conducted for global reading measures of forward fixation count and regressive fixation count

	Forward fixation count			Regressive fixation count		
	*b*	SE	*Z*	*b*	SE	*z*
Intercept	2.16	0.04	**57.72**	0.77	0.06	**12.15**
Dyslexic readers	0.20	0.05	**3.71**	0.54	0.09	**6.20**

*Note*: Significant z values (|z| ≥ 1.96) are marked in **bold.**

**Total reading time**: As predicted, there was a main effect in the reading group whereby dyslexic readers had significantly longer total sentence reading times (Mean 4,252 ms, SD 1813 ms) than the skilled readers (mean 2,872 ms, SD 1151 ms), thus indicating that adults with dyslexia have difficulties with linguistic processing relative to skilled adult readers.

**Saccade amplitude**: There were no significant differences in saccade amplitude for dyslexic readers compared to skilled readers (dyslexic readers Mean 2.58, SD 0.73; skilled readers Mean 2.75, SD 0.77).

**Forward and regressive fixation durations**: Similar to total reading time, there was a main effect of group for both forward fixation duration and regressive fixation duration. Dyslexic readers made longer forward fixations (Mean 236 ms, SD 39 ms) and longer regressive fixations (Mean 227 ms, SD 69 ms) compared to the skilled adult readers (forward Mean 218 ms, SD 35 ms; regressive Mean 207 ms, SD 69 ms).

**Forward and regressive fixation counts**: Dyslexic readers also made significantly more forward fixations (Mean 10.83 SD 3.77) and regressive fixations (Mean 3.96, SD 2.75) compared to skilled readers (forward Mean 8.95, SD 2.46; regressive Mean 2.35, SD 1.61). These additional fixations further contribute to their overall increase in total reading time compared to skilled adult readers.

### 2.3 Local measures

The following measures were analysed for the embedded target words; first fixation duration, single fixation duration, gaze duration, go‐past time, fixation count, and landing position. First fixation duration is the duration of the initial fixation on the target word. Single fixation duration represents those fixations for which the reader made exactly one fixation on the target word during the first pass. Gaze duration is the sum of all forward fixation durations on the target word before the reader leaves that word. Go‐past time is the sum of fixation durations on the target word from when a reader first fixated on that word until their first fixation to the right of that word (including any regressions made before moving forward past the target word). The landing position is the character location on which the eye fixates. Table 5 provides the mean results for first fixation duration, single fixation duration, gaze duration, go‐past time, fixation count, and landing position across the reading group and preview condition. Table 6 provides the LMM outputs and Table 7 provides LMM outputs for the simple effects analysis for when interactions between group and preview occurred.

**TABLE 5:** Average local reading measures

		Identity preview	Transposed	Substituted
First fixation duration (ms)				
	Dyslexia	247 (92)	272 (100)	269 (99)
	Skilled reader	224 (67)	231 (74)	249 (77)
Single fixation duration (ms)				
	Dyslexia	253 (94)	284 (102)	290 (100)
	Skilled reader	225 (67)	237 (75)	257 (77)
Gaze duration (ms)				
	Dyslexia	298 (152)	337 (145)	340 (148)
	Skilled reader	253 (103)	261 (101)	286 (105)
Go‐past time (ms)				
	Dyslexia	406 (383)	434 (280)	478 (343)
	Skilled reader	300 (212)	303 (218)	346 (197)
Landing position (characters)				
	Dyslexia	3.28 (1.59)	3.09 (1.49)	3.10 (1.53)
	Skilled reader	3.51 (1.55)	3.46 (1.61)	3.35 (1.52)
Fixation count				
	Dyslexia	1.85 (1.20)	1.91 (1.07)	2.01 (1.19)
	Skilled reader	1.46 (0.83)	1.43 (0.65)	1.05 (0.71)

*Note*: Mean first fixation duration, single fixation duration, gaze duration, go‐past time, landing position, and fixation count for the target word, as a function of preview condition and reading group. Standard deviations are shown in parentheses.

**TABLE 6:** Model output for LMMs was conducted for local reading measures of first fixation duration (ms), single fixation duration (ms), gaze duration (ms), go‐past time (ms), landing position (characters), and fixation count on identity previews (IP), transposed‐letter previews (TL), and substituted‐letter previews (SL)

	First fixation duration			Single fixation duration			Gaze duration			Go‐past time		
	*b*	SE	*t*	*B*	SE	*T*	*b*	SE	*T*	*b*	SE	*t*
Intercept	5.41	0.02	**236.55**	5.44	0.02	**224.10**	5.52	0.03	**201.08**	5.63	0.04	**153.29**
IP vs. TL	0.03	0.02	1.65	0.05	0.02	**2.81**	0.04	0.02	1.95	0.04	0.03	1.34
TL vs. SL	0.08	0.02	**4.07**	0.09	0.02	**4.49**	0.10	0.02	**4.49**	0.14	0.03	**5.09**
Dyslexic readers	0.09	0.03	**2.80**	0.11	0.03	**3.39**	0.17	0.04	**4.65**	0.28	0.05	**5.80**
IP vs. TL: Dyslexic readers	0.07	0.03	**2.61**	0.07	0.03	**2.52**	0.10	0.03	**3.18**	0.09	0.04	**2.30**
TL vs. SL: Dyslexic readers	−0.09	0.03	**−3.31**	−0.08	0.03	**−2.60**	−0.09	0.03	**−2.79**	−0.06	0.04	−1.45

	Landing position			Fixation count		
	*B*	SE	*t*	*B*	SE	*t*
Intercept	3.46	0.11	**32.40**	0.37	0.04	**9.10**
IP vs. TL	−0.04	0.09	−0.46	−0.02	0.05	−0.38
TL vs. SL	−0.13	0.09	−1.46	0.05	0.05	1.01
Dyslexic readers	−0.29	0.15	**−1.92**	0.27	0.06	**4.87**
IP vs. TL: dyslexic readers	−0.17	0.13	−1.37	0.04	0.07	0.67
TL vs. SL: dyslexic readers	0.16	0.13	1.23	0.01	0.07	0.16

*Note*: Significant *t* values (≥1.96 of standard error, SE) are marked in **bold**.

**TABLE 7:** LMM output for simple effects analysis exploring transposed‐letter previews (TL) compared to substituted‐letter previews (SL) for dyslexic readers in measures of first fixation duration (ms), single fixation duration (ms) and gaze duration (ms)

	First fixation duration			Single fixation duration			Gaze duration		
	*B*	SE	*t*	*B*	SE	*t*	*b*	SE	*T*
Intercept	5.50	0.03	**210.04**	5.57	2.74	**202.69**	5.69	3.13	**182.09**
TL vs. SL	−0.01	0.02	−0.60	1.04	2.36	0.44	10.6	2.38	0.44

*Note*: Significant t values (|t| ≥ 1.96) are marked in **bold**.

**First fixation duration**: For first fixation duration we found the main effect of the group whereby dyslexic readers made longer first fixations on the target word than skilled readers. The main effect of preview whereby identity previews received shorter fixation durations than TL previews was marginally significant (*t* = 1.65). There was a main effect of preview in which TL previews received shorter fixation durations than SL previews. In addition, both interactions were significant (see Table 5). The preview benefit in which identity previews had shorter first fixation durations compared to TL previews was larger for readers with dyslexia than for skilled readers. Furthermore, the TL effect in which TL previews have shorter fixation durations than SL previews only occurred for the skilled adult readers. Thus, whilst skilled adult readers demonstrated the typical benefits of identity previews receiving the shortest fixation durations followed by TL previews and then SL previews requiring the longest fixation durations, dyslexic readers did not demonstrate the same pattern. Dyslexic readers demonstrated a greater benefit of identity previews compared to TL previews. Furthermore, simple effects analysis for the TL effect indicated that the dyslexic readers did not show the typical benefit of TL previews compared to SL previews seen in skilled reading (e.g., Johnson et al., 2007).

**Single fixation duration**: The single fixation results were similar to the first fixation duration results. Dyslexic readers made longer single fixations on the target word than skilled readers. We also found the main effects of previews where identity previews received shorter fixation durations than TL previews and TL previews received shorter fixation durations than SL previews. Both the interactions were again significant; dyslexic readers showed a greater benefit of identity previews compared to TL previews than the skilled readers, and, skilled adult readers showed a greater benefit of TL previews compared to SLs compared to the dyslexic readers. Similar to the first fixation duration, the simple effects analysis indicated that dyslexic readers did not show a significant TL effect (see Table 5).

**Gaze duration**: Dyslexic readers' gaze durations were longer than skilled readers' gaze durations. The main effect for identity previews compared to TL previews was marginally significant (*t* = 1.95), indicating that gaze durations were shorter for identity previews. There was also a significant main effect demonstrating shorter gaze durations following TL previews than SL previews. Both interactions were again significant. Dyslexic readers showed a greater benefit for identity previews compared to TL previews than did skilled readers. The interaction comparing TL previews to SL previews across the reading group was also significant. Although dyslexic readers showed a numerical trend to support a benefit of TL previews compared to SL previews, simple effects analyses indicated that the benefit of TL previews compared to SL previews did not reach significance for dyslexic readers, where the TL effect was larger for skilled adult readers (see Table 5).

**Go‐past time**: Readers with dyslexia had longer go‐past times than skilled readers. There was a main effect in which TL previews resulted in shorter go‐past times compared to SL previews. However, the main effect of identity previews receiving shorter go‐past times than TL previews was not significant, there was, however, a significant interaction whereby dyslexic readers showed a greater benefit for identity previews compared to TL previews than skilled readers. The interaction comparing TL previews to SL previews across the reading group was not significant. By the go‐past time, readers with dyslexia demonstrate a pattern of results more similar to the typical orthographic preview effects shown by skilled readers in early reading measures; whereby identity previews received the shortest viewing durations, followed by TL previews and then SL previews.

**Fixation Count**: There was a significant main effect of group on fixation count; dyslexic adults made more fixations than skilled adult readers. There were no significant effects of the preview manipulation and none of the interactions were significant.

**Landing Position**: There was a marginally significant main effect (*t* = −1.92) of the group on landing position suggesting that dyslexic readers landed earlier into the target word than the skilled adult readers. We found no significant effect of the preview manipulation upon landing position. None of the interactions were significant.

## 3 DISCUSSION

The aim of the experiment was to examine parafoveal processing in dyslexic reading; specifically, to examine parafoveal letter position and letter identity encoding in readers with dyslexia. The pattern of results indicated that both dyslexic and skilled readers gained parafoveal preview benefit during reading; however, when presented with identity previews compared to TL previews, dyslexic readers exhibited a larger parafoveal preview benefit compared to that found for skilled adult readers. We also found that dyslexic readers did not demonstrate the typical TL effect (whereby TL previews provide greater benefit to the reader compared to SL previews) during early reading measures. The TL effect only became significant for dyslexic readers in a later measure of reading that is, go‐past time. Finally, in regard to patterns of foveal eye movements, we found that dyslexic readers required longer viewing durations and made more fixations than skilled readers.

As predicted, both skilled and dyslexic readers were able to gain parafoveal preview benefits during silent sentence reading. Whilst the skilled readers demonstrated the typical pattern of results in which the identity preview condition provided the greatest benefit compared to TL and SL previews, with TL previews providing more benefit than SL previews (Johnson et al., 2007; Pagán et al., 2016; Tiffin‐Richards & Schroeder, 2015), dyslexic readers showed a different pattern of results. In comparison to skilled readers, dyslexic readers showed an increased preview benefit for identity previews compared to TL previews which indicate that dyslexic readers have a greater reliance on initial letter position information for lexical identification than skilled readers. Phonological, semantic, and orthographic information about words are sources of early constraint in word processing to help restrict the number of suitable lexical candidates (Folk & Morris, 1995), and aid in activating the correct lexical representation (Lima & Inhoff, 1985). Dyslexic readers gained greater facilitation from correct orthographic information (and, as such, the correct phonological information) compared to skilled adult readers.

Furthermore, whilst dyslexic readers demonstrated the TL effect during a later reading measure (go‐past time), this effect did not occur in early measures (first fixation duration, single fixation duration, and gaze duration) which, conversely, was the case for skilled readers. This suggests that dyslexic readers do use a flexible letter position encoding mechanism to encode letter position (Johnson et al., 2007; Pagán et al., 2016) but, as they rely more heavily on initial‐letter position information, flexible letter position encoding is less useful and only demonstrated in later reading measures. This result is in line with Jones et al. (2013) who suggested that dyslexic readers have delayed parafoveal preview benefits, and highlights the key role correct orthographic information plays in facilitating the pre‐processing of phonology in dyslexic reading similar to that of child readers in Milledge et al. (2021). In contrast, the orthographically similar previews caused less disruption to pre‐processing in skilled adult reading. Critically, orthographic similarity in the TL previews facilitated lexical activation for skilled readers to a greater extent than it did for dyslexic readers.

In addition to exploring parafoveal processing for readers with dyslexia, we demonstrated the typical differences in eye movement behaviour for dyslexic readers compared to non‐dyslexic readers (e.g., Gangl et al., 2018; Hawelka, Gagl, & Wimmer, 2010; Kirkby, Blythe, Drieghe, & Liversedge, 2011; Kirkby, Webster, Blythe, & Liversedge, 2008). Dyslexic readers required longer viewing durations than the skilled adult readers, made more forward and regressive fixations, and made fixations that landed earlier into the target word compared to skilled readers. This pattern of eye movement behaviour demonstrates dyslexic readers' reading difficulties, providing further evidence that dyslexic readers have slower lexical processing in comparison to skilled readers (Hawelka et al., 2010).

Our results indicate that letter position encoding occurs at a slower rate to letter identity encoding for dyslexic readers (Castles, Davis, Cavalot, & Forster, 2007) during parafoveal processing; dyslexic readers demonstrate parafoveal preview benefits from letter identity information in earlier measures than parafoveal preview benefits from letter position information which occur in later reading measures. Although it occurs at a delayed timeframe, dyslexic readers do encode letter position information flexibly, demonstrating that they are able to use a flexible letter position encoding mechanism.

In regard to dyslexic reading, the current results provide initial evidence that dyslexic readers are able to gain parafoveal preview benefit during silent sentence reading and extend the body of work demonstrating dyslexic readers gain parafoveal preview benefits during RAN (e.g., Jones et al., 2013). Whilst typically, skilled readers are efficient at processing orthographic parafoveal information, dyslexic readers appear to be more dependent on letter position information for lexical activation encoding. Dyslexic readers showed an increased benefit for identity previews compared to TL previews compared to their peers. This increased dependence on letter position information for lexical identification, demonstrated by dyslexic readers, maybe a reading strategy adopted by dyslexic readers. Although many dyslexic readers achieve satisfactorily, or even high levels of reading performance in adulthood (Brunswick et al., 1999), research has suggested that orthographic deficits may remain among adults with dyslexia (e.g., Bruck, 1990, 1992; Brunswick et al., 1999; Elbro et al., 1994; Shaywitz et al., 1999) due to deficient stored orthographic representations (Ehri, 1995; Lemoine et al., 1993; Perfetti, 1992; Swan & Goswami, 1997). Meyler and Breznitz (2005) demonstrated that, compared with skilled adult readers, dyslexic adult readers, had significantly longer reaction times when processing both phonological and orthographic representations of words. As such dyslexic readers may rely more heavily upon correct orthographic input, particularly at the initial‐letter position, to achieve lexical activation.

Recent evidence, however, has shown parafoveal processing of phonology in dyslexic readers comparable to that of their typically developing peers (Blythe et al., 2020), suggesting phonological recoding for readers with dyslexia (i.e., fast, pre‐lexical activation of phonology). However, dyslexic readers require more accurate mapping of letter positions. As such parafoveal pre‐processing would appear to be a skill evident in dyslexic readers, similar to that of skilled adult readers, qualitatively. However, some quantitative differences remain, with dyslexic reading and lexical identification processes being slower and less efficient than those of the skilled adult readers; presumably due to lower quality lexical representations (e.g., Perfetti, 2007).

Whilst the current results provide a detailed account of dyslexic parafoveal processing during silent sentence reading, the parafoveal manipulations were deliberately constrained to the initial letters. As discussed, the initial letters of a word are intrinsically important (Johnson et al., 2007; Johnson & Dunne, 2012; Johnson & Eisler, 2012; Tiffin‐Richards & Schroeder, 2015; White et al., 2008) and this may be due to the requirement for sequential activation of phonological codes to activate phonological representations during reading. Therefore, for dyslexic readers, who have under‐specified lexical representations due to difficulties in mapping phonology to orthography (e.g., Snowling, 1995; Stanovich, 1988), letter position information may be particularly important. The size of the TL effect has been found to be greater when the manipulated letters are internal (29 ms) than when they are external (9 ms; Perea & Lupker, 2003a, 2003b). This is supported by evidence from silent sentence reading, where the cost associated with reading directly fixated TL strings decreased for internal letter manipulations compared with those involving initial or final letters (Johnson, 2007; Johnson & Dunne, 2012; Johnson & Eisler, 2012; White et al., 2008; see also Briihl & Inhoff, 1995; Jordan et al., 2013; Plummer & Rayner, 2012; Rayner et al., 1980; Tiffin‐Richards & Schroeder, 2015). The current findings suggest that disrupting the initial letters of a parafoveally presented word has a greater impact on dyslexic readers who appear to rely heavily upon the correct letter position of these initial letters to aid in lexical activation. It is possible that the increased dependence on letter position information shown by dyslexic adults is specific to these initial letters of the parafoveal word due to the critical role of initial letters with respect to phonological activation (Milledge et al., 2021). Further research is required to determine whether dyslexic readers have a greater reliance on parafoveal letter position information for all letters or specifically for the initial letters of the parafoveal word.

In sum, the current experiment provides a detailed account of the foveal and parafoveal eye movements of dyslexic readers during silent sentence reading. Whilst we provide initial evidence that dyslexic readers do gain orthographic parafoveal preview benefits during reading, we found differences in dyslexic reading across both foveal and parafoveal processing. Importantly, the transpositions of the first and second letters involved in the orthographic manipulation of the previews have a particular cost to the dyslexic readers; suggesting that, dyslexic readers were less able to benefit from correct letter identity information (i.e., in the letter transposition previews) due to the lack of direct mapping of orthography to phonology. The increased disruption the dyslexic readers experienced to their reading times, very early on in processing, could potentially be attributable to the interactive relationship found between orthography and phonology. The pattern of results suggests that the greater overlap in the preview, of orthography and phonology (i.e., in the identity previews), provides greater facilitation to dyslexic parafoveal processing during reading.

## Data Availability

The data that support the findings of this study are openly available in OSF at https://osf.io/45prg/?view_only=21c2c5ce19624c74acb560b7d37c740f
